# 

**Published:** 1871-10

**Authors:** 


					﻿PUBLISHED MONTHLY, AT $3 PER ANNUM, IN ADVANCE.
co
CD
MJ
aS
ki
CD
s
O
nS
*2
aS
P<
CD
5m
P<
4J
O
P
•rM
GJ
5m
cd
P
Of
"S
’S
p«
CD
5m
P<
I_
aS
<D
aS
P
CD
O
O
co
oT
CD
2
o
o
1Q
5m
Q
>
O
5m
cd
<D
aS
CO
<J
P
CD
O
IQ
W
CD
2
o
o
iQ
5m
CD
rd
P
l=>
I
w
o
<
H
O
ATLANTA
Medical and Surgical Journa’.
EDITED BY
J. P. LOGAN, M.D.,
AND
W. F. WESTMORLAND, M.D.,
Professor of the Principles and Practice of Surgery in Atlanta Medical College.
(Pax et Scientia, sed veritas sine timore.
\l
VOLUME IXB-OCTOBER, 1871.-NUMBER 7.
ATLANTA, GEORGIA:
PLANTATION PUBLISHING COMPANY.
1871.
EACH NUMBER CONTAINS SIXTY-FOUR PACES.
hd
c
ui
H
s
GQ
H
W
02
P
•-S
CD
H
CD
P
CD
CO
ft-
CD
p-
e*
o
o
O
'm
£
B-
t?
cd
bi
s»
3
w
'M
<2
<’
S’
0>3
g
p
o
o'
CD
<1
63-
o
P
t?
CD
=->
O
P
»-<
P
£.
K-*
cn
>-«
CD
cd
p-
p
e-t-
cM-
P-
CD
O
9?
o
CD
m
				

